# Long non-coding RNA PVT1 is associated with tumor progression and predicts recurrence in hepatocellular carcinoma patients

**DOI:** 10.3892/ol.2014.2730

**Published:** 2014-11-24

**Authors:** CHAOFENG DING, ZHE YANG, ZHEN LV, CHENGLI DU, HENG XIAO, CHUANHUI PENG, SHAOBING CHENG, HAIYANG XIE, LIN ZHOU, JIAN WU, SHUSEN ZHENG

**Affiliations:** 1 Division of Hepatobiliary and Pancreatic Surgery, Department of Surgery, The First Affiliated Hospital, School of Medicine, Zhejiang University, Zhejiang 310003, P.R. China; 2 Key Laboratory of Combined Multi-Organ Transplantation, Ministry of Public Health, Hangzhou, Zhejiang 310003, P.R. China

**Keywords:** hepatocellular carcinoma, long non-coding RNA, PVT1, recurrence, progression

## Abstract

PVT1, which maps to chromosome 8q24, is a copy number amplification-associated long non-coding RNA. Overexpression of PVT1 is a powerful predictor of tumor progression and patient survival in a diverse range of cancer types. However, the association between PVT1 and hepatocellular carcinoma (HCC) remains unclear. The aim of the present study was to examine the expression pattern of PVT1, and its clinical significance in HCC. Between 2003 and 2012, reverse transcription-quantitative polymerase chain reaction was used to determine the expression levels of PVT1 in two independent cohorts: Cohort one, 58 HCC resection samples; and cohort 2, 214 HCC transplant samples. Additionally, the correlation between PVT1 expression levels and clinical parameters and outcomes was analyzed. The relative expression levels of PVT1 were significantly higher in cancerous tissues compared with the corresponding non-cancerous tissues (cohort one, P=0.0016; cohort two, P=0.0274). Furthermore, overexpression of PVT1 was associated with a higher serum α-fetoprotein expression level (P=0.011) and a higher recurrence rate (P=0.004). Kaplan-Meier analysis indicated that the patients with high PVT1 expression exhibited poor recurrence-free survival (P=0.021), and multivariate analysis demonstrated that high levels of PVT1 expression are an independent predictor for HCC recurrence (P=0.042; hazard ratio, 1.653). Thus, the high expression levels of PVT1 in HCC may serve as a novel biomarker for predicting tumor recurrence in HCC patients, and as a potential therapeutic target.

## Introduction

Hepatocellular carcinoma (HCC) represents the fifth most prevalent malignancy and the second most common cause of cancer-related mortality worldwide, with ~695,900 mortalities per year (1). Half of these cases occur in China, due to high incidence of chronic hepatitis B virus (HBV) infection (2). Liver surgery, including liver resection and liver transplantation, are considered to be curative treatment strategies, as they provide complete oncologic resection (R0 resection). However, tumor recurrence following liver surgery remains a critical issue (recurrence rate, ~30–50%), compromising the long-term survival of patients (3–5). The molecular mechanisms underlying HCC progression remain poorly understood. Therefore, investigating ideal biomarkers for the improved prediction of postoperative recurrence may aid surgeons in selecting patients or adopting preventive strategies for patients at high risk of recurrence.

Previously, RNA sequencing revealed a novel class of transcripts termed long non-coding RNAs (lncRNAs). LncRNAs are >200 nucleotides long and lack protein-coding potential, thus, they were previously regarded as random transcriptional noises. However, increasing evidence has implicated lncRNAs in critical regulatory roles in cancer biology (6–8). LncRNAs have been demonstrated to control gene expression through transcriptional and posttranscriptional regulation (9). Various classic lncRNAs have been characterized in human hepatocarcinogenesis as having oncogenic and tumor suppressive roles, such as HOX transcript antisense RNA (HOTAIR) (10,11), metastasis-associated lung adenocarcinoma transcript 1 (MALAT1) (12), maternally expressed 3 (MEG3) (13) and H19 (14). Our previous studies have demonstrated that overexpression of HOTAIR (10) and MALAT1 (12) exhibit oncogenic properties, and may serve as independent prognostic factors for HCC. Furthermore, MEG3 was frequently downregulated in HCC and inhibited cell growth by functionally interacting with p53 (13). Additionally, H19 suppressed HCC metastasis by epigenetic activation of the microRNA (miR)200 family (14). These data support the hypothesis that MG3 and H19 act as tumor suppressors in HCC.

PVT1, which maps to chromosome 8q24, is a copy number amplification-associated lncRNA. Overexpression of PVT1 is a powerful predictor of tumor progression and patient survival in colorectal (15), ovarian and breast cancers (16). Furthermore, PVT1 exerts regulatory functions in various biological processes, such as proliferation, apoptosis, mobility and invasion (15,16), and chromosome 8q24 is a commonly amplified region in HCC (17,18). However, the association between PVT1 and HCC remains unclear.

The aim of the present study was to examine the expression pattern of PVT1 and its clinical significance in HCC.

## Materials and methods

### Patients and surgical specimens

Fifty-eight snap-frozen HCC tissues and the corresponding non-cancerous tissues were obtained from patients undergoing liver resection at the First Affiliated Hospital of Zhejiang University (Hangzhou, China) between 2009 and 2012 (cohort one). An additional 214 HCC tissues were collected from patients undergoing liver transplantation at the First Affiliated Hospital of Zhejiang University between 2003 and 2012 (cohort two), and were used for survival analysis and validation. The liver tissue specimens were immediately frozen in liquid nitrogen following surgical resection and stored at −80°C prior to the extraction of total RNA. A postoperative histopathological examination by experienced pathologists was used to establish a diagnosis of HCC in these patients. The histological grade of differentiation was evaluated on hematoxylin and eosin-stained sections according to the Edmondson-Steiner grading method (19). Complete clinical and laboratory data were collected in a perspective database. The tumor staging was defined according to the sixth edition of the tumor-node-metastasis (TNM) classification system published by the American Joint Committee on Cancer and the Union for International Cancer Control (20). The requirements for selecting transplant candidates varies depending on the criteria, for example: The Milan criteria defines HCC as a single tumor ≤5 cm or up to three tumors ≤3 cm (21); the University of California, San Francisco (UCSF) criteria defines HCC as a single nodule ≤6.5 cm or up to three nodules ≤4.5 cm, and total tumor diameter ≤8 cm (22); and the Hangzhou criteria defines HCC as a total tumor diameter of ≤8 cm, or a total tumor diameter of >8 cm, with a well or moderately differentiated histopathological grade and a preoperative serum AFP level of ≤400 ng/ml, simultaneously (23).

The present study was conducted with the approval of the Institutional Review Board and Ethics Committee of the First Affiliated Hospital, Zhejiang University (Hangzhou, China) and in accordance with the Declaration of Helsinki. Thus, the study conformed to international and national regulations. Informed consent was obtained from all of the patients.

### Follow-up

Patient follow-up was conducted every 2–3 months during the first two years following surgery and 3–6 months thereafter. The endpoint of study was September 3, 2013. During the follow-up period, all patients were monitored using abdomen ultrasonography, chest X-ray, emission computed tomography and serum AFP tests. Following a suspected recurrence, computed tomography, magnetic resonance imaging or positron emission tomography-computed tomography, were immediately performed. These imaging techniques were required for a new lesion to be detected and recurrence to be diagnosed; an increase in serum AFP levels alone was not regarded as recurrence. Once recurrent tumors were diagnosed, treatment was implemented based on the tumor size, number, location and vascular invasion, as well as the liver function. The recurrence-free survival (RFS) period was calculated from the date of surgery to the date of detection of tumor recurrence, mortality or the most recent observation. All follow-up examinations were performed by two physicians who were unaware of the study. The mean follow-up time was 27.58 months (range, 2–124 months).

### Cell culture

The healthy liver cell line HL7702, the liver cancer cell lines Huh7, SK-hep1, SMMC-7721, HepG2, Hep3B, PLC/PRF/5 and Bel-7402, as well as the metastasis-capable human HCC cell lines MHCC97L, MHCC97H and HCCLM3, were purchased from the American Type Culture Collection (Manassas, VA, USA), the Shanghai Institute of Biochemistry and Cell Biology (Shanghai, China), and the Liver Cancer Institute of Fudan University (Shanghai, China), respectively. All of the cell lines were maintained in Dulbecco’s modified Eagle’s medium with high glucose or RPMI-1640, containing 10% fetal bovine serum, and cultured in a humidified 5% CO_2_ incubator at 37°C.

### RNA extraction and quantitative reverse transcription-quantitative polymerase chain reaction (RT-qPCR)

Total RNAs from the specimens and cell lines were extracted using TRIzol reagent (Invitrogen Life Technologies, Carlsbad, CA, USA); and complementary DNA was synthesized using Moloney murine leukemia virus reverse transcriptase (Promega Corporation, Madison, WI, USA) according to the manufacturer’s instructions. The expression of PVT1 was determined using qPCR, which was performed on an Applied Biosystems 7500 Fast Real-Time PCR system (Applied Biosystems Life Technologies, Foster City, CA, USA) and SYBR^®^ Green dye (Takara Biotechnology Co., Inc., Dalian, China). All PCRs were performed in triplicate and GAPDH was used to normalize mRNA expression levels. Relative quantification was performed using the comparative threshold cycles (2^−ΔΔCt^ method) as described in the manufacturer’s instructions. The primer sequences were as follows: Sense, 3′-CATCCGGCGCTCAGCT-5′ and antisense, 3′-TCATGATGGCTGTATGTGCCA-5′ for PVT1; and sense, 3′-ATGGGGAAGGTGAAGGTCG-5′ and antisense, 3′-GGGGTCATTGATGGCAACAATA-5′ for GAPDH.

### Statistical analysis

Comparisons of continuous data were analyzed using the independent t-test between the two groups, whereas categorical data were analyzed by the χ^2^ test. Comparisons of continuous data among multiple groups were calculated using one-way analysis of variance. A receiver operating characteristic (ROC) curve was used to determine the cut-off value of PVT1 expression that yielded the highest combined sensitivity and specificity for discriminating between patients exhibiting HCC recurrence and those not exhibiting recurrence. Recurrence-free survival was analyzed using the Kaplan-Meier method and compared by performing the log-rank test. Independent prognostic factors were assessed in the univariate and multivariate analysis using the Cox proportional-hazards regression model. All statistical analyses were performed using SPSS for Windows (version 16.0; SPSS, Inc., Chicago, IL, USA) and GraphPad Prism (version 5.0; GraphPad Software, Inc., La Jolla, CA, USA) software. P<0.05 was considered to indicate a statistically significant difference.

## Results

### Characteristics of HCC patients

Snap-frozen HCC tissues were obtained from 272 HCC patients who had undergone liver surgery at the First Affiliated Hospital of Zhejiang University between 2003 and 2012. The present study involved two independent cohorts of HCC patients: Cohort one included 58 HCC patients who had undergone radical resection between 2009 and 2012; while cohort two included 214 HCC patients who had received a liver transplant between 2003 and 2012.

The majority of the HCC patients in the two cohorts were male (91.18%) with a tumor size of >5 cm at the time of surgery (45.96%), an elevated serum AFP level (47.79%) and with tumors exceeding the Milan and UCSF criteria (63.60 and 54.41%, respectively; Table I). In addition, the patients in cohort two were HCC transplant patients, thus representing a group of patients with advanced disease. Furthermore, the patients in cohort two were relatively younger than those in cohort one, and more patients in cohort two exhibited HBV infection, liver cirrhosis, multifocal tumor, portal vein tumor thrombosis (PVTT) and advanced TNM stage.

### PVT1 overexpression in the two independent HCC cohorts and liver cancer cell lines

**Figure 1:**
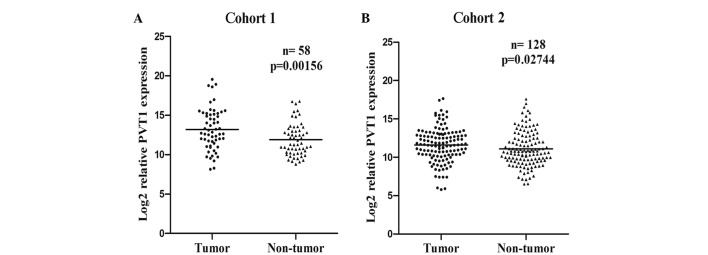
PVT1 expression levels in hepatocellular carcinoma tumor (HCC) tissue and the corresponding non-tumorous tissue. PVT1 expression levels were normalized to an internal control and log2-transformed. (A) Cohort one consists of 58 paired HCC resection samples and (B) cohort two consists of 128 paired HCC transplant samples. Horizontal lines in the plots represent the mean values. P-values between samples were obtained by performing a paired t-test.

**Figure 2:**
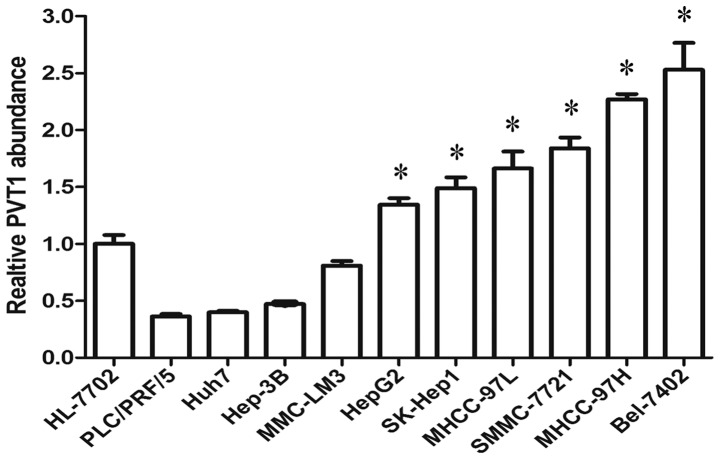
Relative PVT1 expression levels in 10 liver cancer cell lines. HL-7702 is a healthy liver cell line. Data are the mean ± standard error of the mean (n=3). ^*^P<0.05, vs. the control (HL-7702 cells), according to the paired t-test.

In the present study, the expression levels of PVT1 in the 272 HCC patients from the two independent cohorts were determined using RT-qPCR. Compared with the corresponding non-tumor liver tissues of the HCC cohorts, PVT1 expression was significantly increased in the cancerous tissues of the patients in cohort one (P=0.0016; Fig. 1A) and cohort two (P=0.0274; Fig. 1B). Furthermore, the expression of PVT1 was defined in 10 liver cancer cell lines and one healthy liver cell line. Of the 10 liver cancer cell lines, six (HepG2, SK-Hep1, MHCC-97L, SMMC-7721, MHCC-97H and Bel-7402) expressed a higher level of PVT1 than the healthy liver cell line (Fig. 2). Thus, the present study determined that PVT1 expression was significantly increased in HCC.

### Association between PVT1 and HCC progression

**Figure 3:**
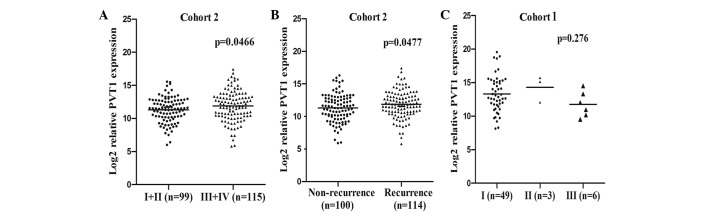
PVT1 expression levels defined according to the tumor-node-metastasis stage and recurrence status. (A) Cohort two: Early (stages I + II) vs. advanced stage (stages III + IV). (B) Cohort two: Non-recurrence vs. recurrence. (C) Cohort one: Three different stages (I vs. II vs. III). PVT1 expression levels were normalized to an internal control and log2-transformed. Horizontal lines in the plots represent the mean values. P-values were analyzed using (A and B) the independent t-test and (C) one-way analysis of variance.

As PVT1 expression was significantly increased in HCC, the association between PVT1 expression in HCC and disease progression was evaluated. First, the expression level of PVT1 in different TNM-stage patients was assessed, as TNM staging is a widely accepted system for HCC stratification. In HCC cohort two, advanced-stage patients (stages III and IV; n=115) exhibited increased expression levels of PVT1 compared with early-stage patients (stages I and II; n=99) (P=0.0466; Fig. 3A). Additionally, the patients exhibiting disease recurrence (n=114) demonstrated higher levels of PVT1 expression compared with the non-recurrence recipients (n=100) (P=0.0477;Fig. 3B). However, in HCC cohort one, it was difficult to analyze the data with sufficient statistical power due to the limited number of patients with an advanced disease stage (Fig. 3C).

To investigate the clinicopathological correlation of PVT1 expression in HCC tissues, the patients were divided into high and low expression groups according to the cut-off value obtained from the ROC curve analysis. No significant correlations with any of the clinicopathological parameters tested were observed in HCC cohort one (Table II). In HCC cohort two, no significant correlations were identified between PVT1 expression and clinicopathological parameters such as age, gender, tumor number, tumor size, PVTT, histopathological grade or TNM stage (Table III). However, high PVT1 expression levels in HCC cohort two were significantly correlated with a higher AFP level (P=0.011) and higher recurrence rate (P=0.004) (Table III). Thus, these data indicate that high PVT1 expression levels in HCC may be associated with disease progression.

### PVT1 predicts HCC recurrence following liver transplantation

**Figure 4:**
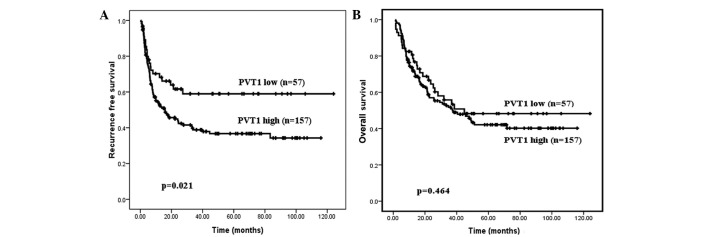
Kaplan-Meier survival curves according to PVT1 level. Patients with high PVT1 expression had poor (A) recurrence free survival and (B) overall survival rates. P-values were calculated using the log-rank test.

To determine whether PVT1 could be employed as a prognostic biomarker for HCC, clinical data of HCC cohort two were analyzed in detail. Using the cut-off value, the 214 patients were divided into two groups: A low-expression group (n=57) and a high-expression group (n=157). Kaplan-Meier analysis indicated that the patients with high PVT1 expression levels had a poor RFS period (P=0.021; Fig. 4A). Although no statistical significance of PVT1 expression as a predictor of overall survival was determined, patients exhibiting high PVT1 expression levels demonstrated a trend for poor prognoses (Fig. 4B; P=0.464).

To identify the risk factors associated with post-transplant RFS, 11 clinicopathological factors were evaluated by performing Cox univariate and multivariate analyses. Univariate analysis demonstrated that the significant prognostic factors for HCC recurrence were tumor size, tumor number, histopathological grade, PVTT, preoperative AFP level, TNM stage and PVT1 expression (all P<0.05). Only tumor size (HR, 2.462; 95% CI, 1.652–3.671; P<0.001), tumor number (HR, 1.802; 95% CI, 1.194–2.719; P=0.005), PVTT (HR, 2.075, 95% CI, 1.418–3.037; P<0.001), preoperative AFP level (HR, 1.539;95% CI, 1.027–2.305; P=0.037) and PVT1 expression (HR, 1.653; 95% CI, 1.019–2.681; P=0.042) were identified as independent prognostic factors associated with tumor recurrence following liver transplantation, as determined by the Cox multivariate analysis (Table IV).

## Discussion

The complexity of the human transcriptome has been highlighted by various high-throughput studies (24,25); ≤70% of the genome is transcribed into RNA that does not act as templates for protein (26). The non-protein-coding portion of the genome constitutes the vast majority of genomic information, as well as exhibiting critical functional roles (27). Improved understanding of the aberrant expression patterns, cellular functions and underlying mechanisms of the non-protein-coding genome may aid in expanding the current understanding of the complex regulatory network in cancer biology.

Although the majority of previous studies have focused on short RNAs in cancer research, such as miRNAs, lncRNAs are gaining prominence. A number of classic lncRNAs have been implicated in human hepatocarcinogenesis, exhibiting oncogenic or tumor suppressive roles. One such example of oncogenic lncRNA is HOTAIR, which was initially identified in foreskin fibroblasts. HOTAIR resides in the HOX C locus, acting as a modular scaffold to recruit the polycomb repressive complex 2 to specific target sequences that ultimately results in the suppression of numerous genes (28). Our previous study demonstrated that the expression of HOTAIR is upregulated in HCC tissues compared with paired non-cancerous tissues, and high expression levels of HOTAIR were an independent prognostic marker for HCC recurrence and shorter survival (10). An additional classic oncogenic lncRNA is MALAT1, which regulated the alternative splicing of a subset of pre-mRNAs to promote cancer metastasis (29). In our previous study, MALAT1 was unregulated in HCC and served as a negative prognostic factor for tumor progression and patient survival (12).

Furthermore, tumor suppressive lncRNAs may affect various tumor suppressor pathways. For example, MEG3 was identified to be frequently downregulated in HCC by miR29a-mediated promoter methylation, and MEG3 inhibited cell growth by functionally interacting with the p53 signaling pathway (13). Additionally, H19 associated with the protein complex heterogeneous nuclear ribonucleoprotein U/P300/cAMP-response element binding protein-associated factor/RNA polymerase II, epigenetically activating the miR-200 family, and thus suppressing HCC metastasis (14).

Various reports have presented evidence that PVT1 contributes to cancer pathophysiology. For example, PVT1 was markedly overexpressed in colorectal (15), ovarian and breast cancers (16). In the current study, PVT1 expression was initially analyzed in 58 pairs of HCC resection samples (cohort one), and it was identified that PVT1 was significantly upregulated in HCC. Subsequently, the PVT1 expression level from an independent cohort of 214 HCC patients (cohort two) was analyzed. The upregulation of PVT1 was validated in this independent cohort. The findings of the present study indicate that PVT1 is predominantly overexpressed in HCC tissues, regardless of the type of surgical intervention that the patients undergo. However, the molecular mechanism underlying the upregulation of PVT1 remains poorly understood. Guan *et al* (16) demonstrated that amplification of chromosome 8q24 increased the expression of PVT1 in ovarian and breast cancers. Consistent with this finding, Takahashi *et al* (15) identified that chromosome 8q24 copy number gain promoted the expression of PVT1 in colorectal cancer. Additional studies are required to determine if PVT1 is similarly regulated in HCC.

Furthermore, the present study identified that PVT1 was more likely to be overexpressed in advanced-stage and recurrence patients. Correlation analysis indicated that increased expression of PVT1 was associated with a higher AFP level and a higher recurrence rate. These data support the hypothesis that PVT1 is associated with disease progression. In addition, survival analysis demonstrated that the patients with high PVT1 expression exhibited poor RFS. Multivariate analysis identified that PVT1 was an independent prognostic factor for RFS. Therefore, data from the present study indicate that PVT1 may be a novel biomarker for risk surveillance and adjuvant therapy screening of HCC patients following liver transplantation. Furthermore, overexpression of PVT1 may be used by surgeons to identify high-risk patients who may benefit from preventive strategies as opposed to surgery.

The effects and precise molecular mechanisms underlying the altered expression of PVT1 in HCC are unclear. Guan *et al* (16) demonstrated that depletion of PVT1 may decrease cell proliferation and increase cell apoptosis in ovarian and breast cancer cell lines. Similarly, Takahashi *et al* (15) identified that PVT1 knockdown promotes apoptosis in colorectal cancer cell lines via the TGF-β signaling pathway. Thus, the detailed mechanism is likely to be complex. Barsotti *et al* (30) identified PVT1 as a p53-inducible target gene. Furthermore, the PVT1 locus produced numerous spliced non-coding RNAs, as well as six miRNAs, which may be a molecular switch for cell life and death. Further experiments are required to elucidate the detailed interplay.

In conclusion, the present study demonstrated that PVT1 was overexpressed in two independent human HCC cohorts and 10 liver cancer cell lines. Increased expression levels of PVT1 were associated with a higher AFP level and a higher recurrence rate. Furthermore, PVT1 served as an independent prognostic factor for RFS. Thus, the findings of the present study indicate that PVT1 may act as a novel biomarker for predicting tumor recurrence in HCC patients and may be a potential therapeutic target.

## Figures and Tables

**Table I:** Clinical characteristics of HCC patients.

	Cohort one (n=58)	Cohort two (n=214)	P-value
Age, years			0.000^a^
Median	60	49	
Range	26–86	20–71	
Gender, n (%)			0.951
Female	5 (8.62)	19 (8.88)	
Male	53 (91.38)	195 (91.12)	
HBV, n (%)			0.000^a^
Negative	11 (18.97)	6 (2.34)	
Positive	47 (81.03)	209 (97.66)	
Cirrhosis, n (%)			0.000^a^
No	22 (37.93)	2 (0.93)	
Yes	36 (62.07)	212 (99.07)	
Tumor size, n (%)			0.059
≤5 cm	25 (43.10)	122 (57.01)	
>5 cm	33 (56.90)	92 (42.99)	
Tumor number, n (%)			0.000^a^
=1	52 (89.66)	89 (41.59)	
>1	6 (10.34)	125 (58.41)	
PVTT, n (%)			0.000^a^
Negative	54 (93.10)	142 (66.36)	
Positive	4 (6.90)	72 (33.64)	
AFP, n (%)			0.270
≤400 ng/ml	34 (58.62)	108 (50.47)	
>400 ng/ml	24 (41.38)	106 (49.53)	
Histopathological grade, n (%)			0.512
Well + moderately	27 (46.55)	110 (51.40)	
Poorly	31 (53.45)	104 (48.60)	
TNM stage, n (%)			0.000^a^
I + II	52 (89.66)	99 (46.26)	
III + IV	6 (10.34)	115 (53.74)	
Milan criteria (19), n (%)			0.374
Within criteria	24 (41.38)	75 (35.05)	
Beyond criteria	34 (58.62)	139 (64.95)	
UCSF criteria (20), n (%)			0.175
Within criteria	31 (53.45)	93 (43.46)	
Beyond criteria	27 (46.55)	121 (56.54)	
Hangzhou criteria (21), n (%)			0.000^a^
Within criteria	46 (79.31)	105 (49.07)	
Beyond criteria	12 (20.69)	109 (50.93)	

a P<0.05 was considered to indicate a statistically significant difference, using the independent samples t-test for age and the χ^2^ test for all other data.

HCC, hepatocellular carcinoma; HBV, hepatitis B virus; PVTT, portal vein tumor thrombosis; AFP, serum α-fetoprotein; TNM, tumor-node-metastasis; UCSF, University of California, San Francisco.

**Table II:** Clinicopathological correlation of PVT1 expression in human HCC (cohort one).

		Low PVT1 expression		High PVT1 expression		
				
Factors	Cases, n	n	%	n	%	P-value
Age, years						
≤60	33	5	55.56	28	57.14	0.930
>60	25	4	44.44	21	42.86	
Gender						
Female	5	0	0.00	5	10.20	0.316
Male	53	9	100.00	44	89.80	
HBV						
Negative	11	1	11.11	10	20.41	0.513
Positive	47	8	88.89	39	79.59	
Cirrhosis						
No	22	4	44.44	18	36.73	0.661
Yes	36	5	55.56	31	63.27	
Tumor size, cm						
≤5	25	2	22.22	23	46.94	0.169
>5	33	7	77.78	26	53.06	
Tumor number						
Single	52	8	88.89	44	89.80	0.935
Multiple	6	1	11.11	5	10.20	
PVTT						
Absent	54	8	88.89	46	93.88	0.587
Present	4	1	11.11	3	6.12	
Preoperative AFP level, ng/ml						
≤400	34	7	77.78	27	55.10	0.204
>400	24	2	22.22	22	44.90	
Histopathological grade						
Well + moderately	27	5	55.56	22	44.90	0.556
Poorly	27	4	44.44	27	55.10	
TNM stage						
I + II	52	7	77.78	45	91.84	0.203
III + IV	6	2	22.22	4	8.16	
Milan criteria (21)						
Within criteria	24	2	22.22	22	44.90	0.204
Beyond criteria	34	7	77.78	27	55.10	
UCSF criteria (22)						
Within criteria	31	3	33.33	28	57.14	0.188
Beyond criteria	27	6	66.67	21	42.86	
Hangzhou criteria (23)						
Within criteria	46	6	66.67	40	81.63	0.308
Beyond criteria	12	3	33.33	9	18.37	

a P<0.05 was considered to indicate statistical significance a statistically significant difference, according to the χ^2^ test.

HCC, hepatocellular carcinoma; HBV, hepatitis B virus; PVTT, portal vein tumor thrombosis; AFP, α-fetoprotein; TNM, tumor-node-metastasis; UCSF, University of California, San Francisco.

**Table III:** Clinicopathological correlation of PVT1 expression in human HCC (cohort two).

		Low PVT1 expression		High PVT1 expression		
				
Factor	Cases, n	n	%	n	%	P-value
Age, years						
≤60	187	49	85.96	138	87.90	0.707
>60	27	8	14.04	19	12.10	
Gender						
Female	19	4	7.02	15	9.55	0.564
Male	195	53	92.98	142	90.45	
HBV						
Negative	5	2	3.51	3	1.91	0.494
Positive	210	55	96.49	154	98.09	
Cirrhosis						
No	2	1	1.75	1	0.64	0.453
Yes	212	56	98.25	156	99.36	
Tumor size, cm						
≤5	122	33	57.89	89	56.69	0.875
>5	92	24	42.11	68	43.31	
Tumor number						
Single	89	28	49.12	61	38.85	0.178
Multiple	125	29	50.88	96	61.15	
PVTT						
Absent	142	40	70.18	102	64.97	0.476
Present	72	17	29.82	55	35.03	
Preoperative AFP level, ng/ml						
≤400	108	37	64.91	71	45.22	0.011^a^
>400	106	20	35.09	86	54.78	
Histopathological grading						
Well + moderately	110	35	61.40	75	47.77	0.078
Poorly	104	22	38.60	82	52.23	
TNM stage						
I + II	99	30	52.63	69	43.95	0.260
III + IV	115	27	47.37	88	56.05	
Recurrence						
No	100	36	63.16	64	40.76	0.004^a^
Yes	114	21	36.84	93	59.24	
Milan criteria (19)						
Within criteria	75	24	42.11	51	32.48	0.192
Beyond criteria	139	33	57.89	106	67.52	
UCSF criteria (20)						
Within criteria	93	27	47.37	66	42.04	0.487
Beyond criteria	121	30	52.63	91	57.96	
Hangzhou criteria (21)						
Within criteria	105	31	54.39	74	47.13	0.348
Beyond criteria	109	26	45.61	83	52.87	

a P<0.05 was considered to indicate a statistically significant difference, according to the χ^2^ test.

HCC, hepatocellular carcinoma; HBV, hepatitis B virus; PVTT, portal vein tumor thrombosis; AFP, serum α-fetoprotein; TNM, tumor-node-metastasis; UCSF, University of California, San Francisco.

**Table IV:** Cox univariate and multivariate analysis of predictors of recurrence in hepatocellular carcinoma patients following liver transplant.

	Univariate analysis			Multivariate analysis		
		
Variable for tumor recurrence	HR	95% CI	P-value	HR	95% CI	P-value
Age, years (>60 vs. ≤60)	0.669	0.349–1.280	0.224			
Gender (male vs. female)	1.577	0.768–3.239	0.214			
HBV	21.091	0.159–2793	0.221			
Cirrhosis	1.039	0.145–7.443	0.970			
Tumor size, cm (>5 vs. ≤5)	3.431	2.340–5.029	0.000	2.462	1.652–3.671	0.000^a^
Tumor number (multiple vs. single)	2.393	1.597–3.582	0.000	1.802	1.194–2.719	0.005^a^
Histopathological grade (poorly vs. well + moderately)	1.665	1.148–2.415	0.007			
PVTT (present vs. absent)	2.826	1.947–4.102	0.000	2.075	1.418–3.037	0.000^a^
Preoperative AFP level, ng/ml (>400 vs. ≤400)	2.380	1.622–3.492	0.000	1.539	1.027–2.305	0.037^a^
TNM stage (III + IV vs. I + II)	4.584	2.987–7.034	0.000			
PVT1 expression (high vs. low)	1.738	1.082–2.792	0.022	1.653	1.019–2.681	0.042^a^

a P<0.05

HR, hazard ratio; CI, confidence interval; HBV, hepatitis B virus; PVTT, portal vein tumor thrombosis; AFP, α-fetoprotein; TNM, tumor-node-metastasis.
